# Supplementary material for: Measured intrapatient radiomic variability as a predictor of treatment response in multi-metastatic soft tissue sarcoma patients
Source: medRxiv. 2025 May 14:2025.04.11.25325700. Originally published 2025 Apr 13. Preprint. [Version 2] doi: 10.1101/2025.04.11.25325700 (PMC12036393; doi:10.1101/2025.04.11.25325700)
Supplement: Supplement 1 [file NIHPP2025.04.11.25325700v2-supplement-1.pdf]

medRxiv preprint doi: https://doi.org/10.1101/2025.04.11.25325700; this version posted May 14, 2025. The copyright holder for this preprint (which was not certified by peer review) is the author/funder, who has granted medRxiv a license to display the preprint in perpetuity.
It is made available under a CC-BY 4.0 International license .

Supplemental

Number of Features	SUBSET		
	Volumetric Response	Liquid Biopsy	Survival
Total Extracted	1218		
High Variance, Uncorrelated to Volume	248	265	261
High Variance, Uncorrelated to Volume and Other Features	16	10	13

Table S1: *Summary of radiomic features used to calculate MIRV for each subset of the patient data: volumetric response, liquid biopsy and survival. Numbers of features used for the calculation are recorded at different stages of the unsupervised feature reduction step.*

Radiomics Features			SUBSET		
Filter	Class	Name	Volumetric Response	Liquid Biopsy	Survival
Gradient	GLSZM	SmallAreaEmphasis			
Wavelet-HHL	GLCM	ClusterShade			
Wavelet-HHL	FirstOrder	Median			
Wavelet-HHH	FirstOrder	Median			
SquareRoot	GLCM	ClusterShade			
Wavelet-HLH	FirstOrder	Median			
Wavelet-LHH	FirstOrder	Median			
Wavelet-LLH	GLCM	ClusterShade			
Exponential	GLSZM	SmallAreaEmphasis			
Wavelet-HLH	GLSZM	SmallAreaEmphasis			
Wavelet-LHL	GLCM	ClusterShade			
Wavelet-LLL	GLCM	Correlation			
Wavelet-HLH	GLCM	ClusterShade			
Wavelet-HLL	GLCM	ClusterShade			

medRxiv preprint doi: https://doi.org/10.1101/2025.04.11.25325700; this version posted May 14, 2025. The copyright holder for this preprint
(which was not certified by peer review) is the author/funder, who has granted medRxiv a license to display the preprint in perpetuity.
It is made available under a CC-BY 4.0 International license .

Wavelet-LHH	GLCM	ClusterShade			
Wavelet-HLL	FirstOrder	Kurtosis			
Wavelet-LLH	GLCM	Correlation			
Wavelet-HHH	GLSZM	SmallAreaEmphasis			
Square	GLSZM	SmallAreaEmphasis			
Logarithm	FirstOrder	InterquartileRange			
Logarithm	GLDM	LargeDependenceHighGrayLevelEmphasis			
Wavelet-LHH	GLSZM	SmallAreaEmphasis			

Table S2: *Summary of radiomic features used to calculate MIRV for each subset of the patient data: volumetric response, liquid biopsy and survival. Five features are used in all three subsets, and sixteen features are used by at least two subsets. Five features are unique to the volumetric response subset and three features are unique to the liquid biopsy subset.*

Variable	**Hazard Ratio [95% conf.int.]**	**z**	**p**
Patient Age >= 65	0.88 [0.50,1.56]	-0.43	0.67
ECOG Performance Status	1.46 [0.84,2.53]	1.35	0.18
Histologic classification	1.82 [1.44,2.31]	4.94	<0.005
MIRV (max) Dissimilarity	1.21 [0.62,2.38]	0.55	0.58
MIRV (max) Distance	1.00 [0.96,1.05]	0.07	0.94
Baseline Volume (total)	1.57 [1.21,2.05]	3.35	<0.005
RECIST	0.54 [0.40,0.72]	-4.08	<0.005
ECOG x MIRV Dissimilarity	1.26 [0.68,2.37]	0.73	0.46
Histology x MIRV Dissimilarity	0.68 [0.52,0.87]	-3.04	<0.005
Age x MIRV Dissimilarity	1.18 [0.63,2.21]	0.51	0.61
RECIST x MIRV Dissimilarity	1.14 [0.82,1.59]	0.79	0.43

Table S3: *Hazard ratios (HR) with 95% confidence intervals (CI), z-scores, and p-values for key clinical and radiomic variables in a multivariable Cox proportional hazards model assessing overall survival in metastatic soft-tissue sarcoma patients. Significant predictors of worse survival include histologic classification (HR = 1.82, $p < 0.005$), and baseline tumor volume (HR = 1.57, $p < 0.005$). RECIST response was significantly associated with better survival (HR = 0.54, $p < 0.005$). MIRV-based metrics (dissimilarity and distance) were not significant prognostic factors ($p = 0.58$ and $p = 0.94$, respectively), but the histology-MIRV interaction term (Dissimilarity) was significantly associated with better survival (HR = 0.68, $p < 0.005$).*
